# Promotion of Protein Solubility and Reduction in Stiffness in Human Lenses by Aggrelyte-1: Implications for Reversing Presbyopia

**DOI:** 10.3390/ijms24032196

**Published:** 2023-01-22

**Authors:** Sudipta Panja, Hanmant Gaikwad, Johanna Rankenberg, Mi-Hyun Nam, Ram H. Nagaraj

**Affiliations:** 1 Department of Ophthalmology, Sue Anschutz-Rodgers Eye Center, School of Medicine, Aurora, CO 80045, USA; 2 Department of Pharmaceutical Sciences, Skaggs School of Pharmacy and Pharmaceutical Sciences, University of Colorado, Anschutz Medical Campus, Aurora, CO 80045, USA

**Keywords:** lens protein solubility, disulfide reduction, acetylation, elasticity, stiffness, presbyopia

## Abstract

With aging, human lenses lose the ability to focus on nearby objects due to decreases in accommodative ability, a condition known as presbyopia. An increase in stiffness or decrease in lens elasticity due to protein aggregation and insolubilization are the primary reasons for presbyopia. In this study, we tested aggrelyte-1 (*S*,*N*-diacetyl glutathione diethyl ester) for its ability to promote protein solubility and decrease the stiffness of lenses through its dual property of lysine acetylation and disulfide reduction. Treatment of water-insoluble proteins from aged human lenses (58–75 years) with aggrelyte-1 significantly increased the solubility of those proteins. A control compound that did not contain the S-acetyl group (aggrelyte-1C) was substantially less efficient in solubilizing water-insoluble proteins. Aggrelyte-1-treated solubilized protein had significant amounts of acetyllysine, as measured by Western blotting and LC-MS/MS. Aggrelytes increased the protein-free thiol content in the solubilized protein. Aged mouse (7 months) and human (44–66 years) lenses treated with aggrelyte-1 showed reduced stiffness accompanied by higher free thiol and acetyllysine levels compared with those treated with aggrelyte-1C or untreated controls. Our results suggested that aggrelyte-1 reduced lens stiffness through acetylation followed by disulfide reduction. This proof-of-concept study paves the way for developing aggrelyte-1 and related compounds to reverse presbyopia.

## 1. Introduction

Presbyopia, a gradual, age-related loss of the ability to focus actively on nearby objects, is a major vision-impeding problem for people over 50 years of age. In 2015, approximately 1.8 billion people globally had presbyopia; nearly 826 million had visual impairment because of the absence of adequate vision correction [1]. The prevalence of presbyopia in the US ranges from 83.0% to 88.9% for adults aged ≥45 years [2]. Presbyopia causes enormous productivity loss, estimated to be approximately 25 billion dollars a year [3], due to uncorrected vision and has a negative impact on quality of life. Thus, developing methods for preventing or postponing the onset or reversing presbyopia is urgently needed.

Age-associated hardening (stiffening) and the consequential loss of lens elasticity are significant contributors to presbyopia. The hardening of the lens occurs concurrently with an increase in the water-insoluble protein content. Biochemical mechanisms that promote protein insolubilization through covalent and hydrophobic interactions appear to be players in presbyopia. Because of the negligible turnover of proteins, chemical modifications that accrue in lens proteins have been considered factors for protein insolubilization and stiffening in the lens [4]. The major chemical protein modifications are sulfhydryl oxidation, deamidation, advanced glycation end product formation, truncation, and racemization [5,6,7,8,9,10]. In aging lens proteins, sulfhydryl oxidation occurs due to increased oxidative stress [11,12]. Most of the cysteine residues in lens proteins are oxidized by the age of 60–65 years in humans [13], and oxidized proteins are present mainly in the water-insoluble portion of aged lenses. Several strategies have been employed to reduce disulfide bonds in lens proteins to reverse presbyopia [14]. One such is the use of the choline ester of lipoic acid [14], which has shown an improvement in vision upon the topical application in a human clinical trial [15]. Furthermore, a recent study showed that lipoic acid could reverse oxidative stress-induced stiffness in porcine lenses [16]. Although disulfide formation in lens proteins is probably a significant contributor to protein aggregation and insolubilization, other chemical modifications (listed above) are also likely to participate.

**Figure 1:**
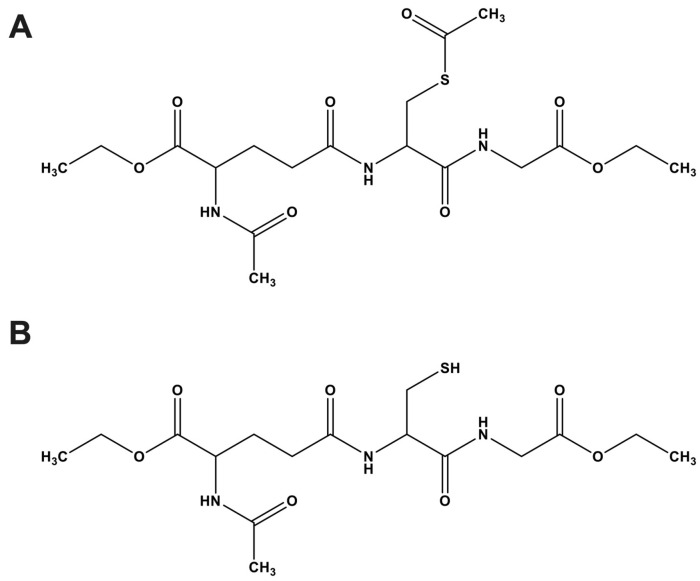
Structures of aggrelyte-1 and 1C. Structure of aggrelyte-1 (*S*,*N*-diacetyl glutathione diethyl ester) (**A**) and aggrelyte-1C (N-acetyl glutathione diethyl ester) (**B**).

Acylation is another significant protein modification in human lenses [17]. Acylation adds an acyl group to the epsilon amino group of lysine residues in proteins [18,19]. Such addition occurs mainly through the nonenzymatic addition of an acetyl group by acetyl-CoA [18,19]. Enzymatic acetylation catalyzed by lysine acetyltransferase can also contribute to protein acetylation [18]. We have previously demonstrated that acetylation, succinylation, propionylation, and malonylation occur in human lenses and that acetylation is dominant among these modifications [20,21,22]. We have also shown that the absence of sirtuin-3 and -5 promotes the acetylation of lens proteins in mice [23]. Furthermore, we have demonstrated that acetylation enhances the chaperone activity of αB-crystallin [24]. In this study, we report the findings on acetylation and disulfide reduction by the glutathione derivatives *S*,*N*-diacetyl glutathione diethyl ester (aggrelyte-1, Figure 1A) and N-acetyl glutathione diethyl ester (aggrelyte-1C, Figure 1B) and their ability to solubilize water-insoluble proteins of the human lens and reduce lens stiffness in aged mouse lenses.

## 2. Results

### 2.1. Stability of Aggrelytes under Physiological Conditions

Before we tested aggrelytes for their ability to increase protein solubility and reduce lens stiffness, we determined their stability at pH 7.4 and 37 °C. After withdrawing aliquots at 0 (immediately after incubation), 3, and 7 days, we lyophilized the solution, and d^6^-DMSO was added for NMR spectroscopy. The NMR data suggested that the S-acetyl bond of aggrelyte-1 was somewhat unstable. The remaining amount of aggrelyte-1 was 58% and 45% after 3 and 7-day incubation periods, respectively. The remaining amount of aggrelyte-1C was 97.7% and 90.2%, respectively (Figure S1).

### 2.2. Aggrelyte-1 Solubilizes Water-Insoluble Lens Proteins (WI) and Increases the AcK Modification in Solubilized Proteins of Aged Human Lenses

**Figure 2:**
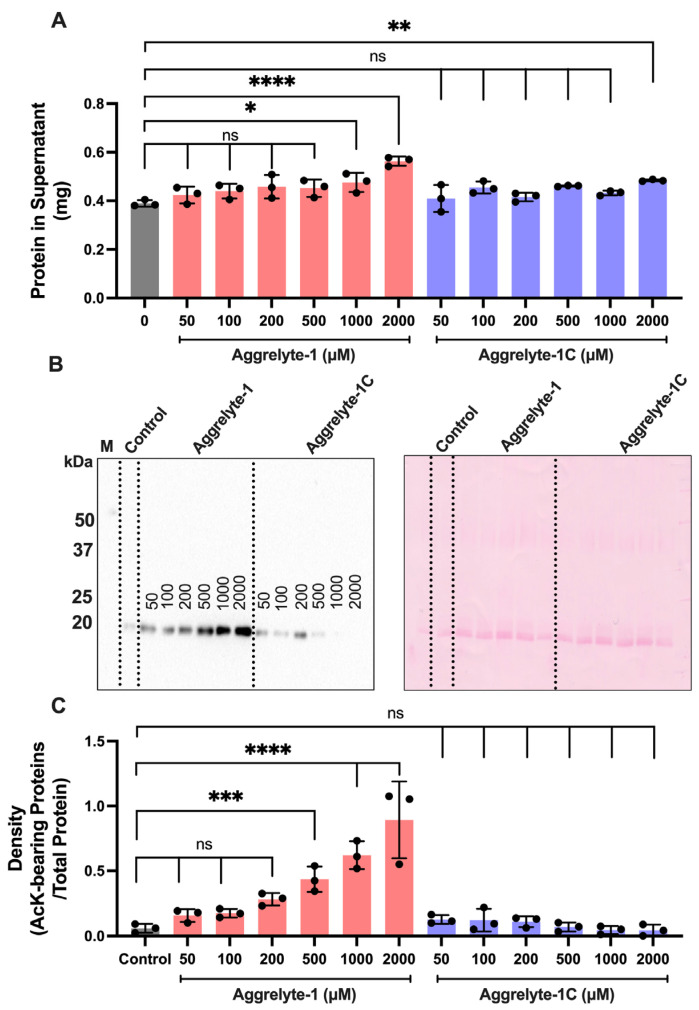
Aggrelyte concentration-dependent solubilization and acetylation of human lens WI. Two milligrams of WI from a human lens (75 years) were suspended in 0.4 mL of 50 mM phosphate buffer, pH 7.4, and the suspension was treated with varying concentrations of aggrelyte-1 or aggrelyte-1C (0–2000 µM) for 24 h at 37 °C. The suspension was centrifuged at 20,000× *g* for 20 min at 4 °C, and the soluble protein content in the supernatant was determined using the BCA assay (**A**). For each concentration of aggrelytes, the samples were separately processed three times and analyzed. The level of AcK was determined for each sample by Western blotting (**B**). Five micrograms of protein from each sample were used for Western blotting. The ponceau S-stained membrane showed protein loading. The bar graphs represent the density plot of triplicate measurements (mean ± S.D.) (**C**). * *p* < 0.05, ** *p* < 0.01, *** *p* < 0.001, **** *p* < 0.0001, ns = not significant.

Two milligrams of WI from a human lens (75 years) were suspended in 0.4 mL of 50 mM phosphate buffer, pH 7.4, and the suspension was treated with increasing concentrations of aggrelyte-1 or aggrelyte-1C (0–2000 µM) for 24 h at 37 °C. The suspension was centrifuged, and the protein (WS) content in the supernatant was determined. The control samples without aggrelytes showed 0.39 mg/0.4 mL soluble protein after 24 h incubation. The results (Figure 2A) indicated a concentration-dependent increase in the solubility of WI with aggrelytes. We did not observe a significant difference in the solubilized protein between aggrelyte-1 and aggrelyte-1C up to 500 µM. However, the samples treated with 1000 µM and 2000 µM aggrelyte-1 showed significantly higher solubilized protein content than the aggrelyte-1C-treated samples. The samples treated with 1000 µM aggrelyte-1 and aggrelyte-1C had 0.48–0.51 and 0.42–0.44 mg solubilized proteins, whereas those treated with 2000 µM aggrelyte-1 and aggrelyte-1C had 0.54–0.58 and 0.48–0.49 mg proteins. We determined the AcK content in solubilized proteins by Western blotting. An aggrelyte concentration-dependent increase in AcK content was observed in the solubilized protein (Figure 2B,C). The control samples showed AcK bands around the 20 kDa region, representing an in situ modification of crystallins [20]. In the aggrelyte-1C-treated samples, the AcK levels were not significantly different from those in the controls. Based on the effect on solubility and level of acetylation, we used 2000 µM aggrelytes in all subsequent experiments.

**Figure 3:**
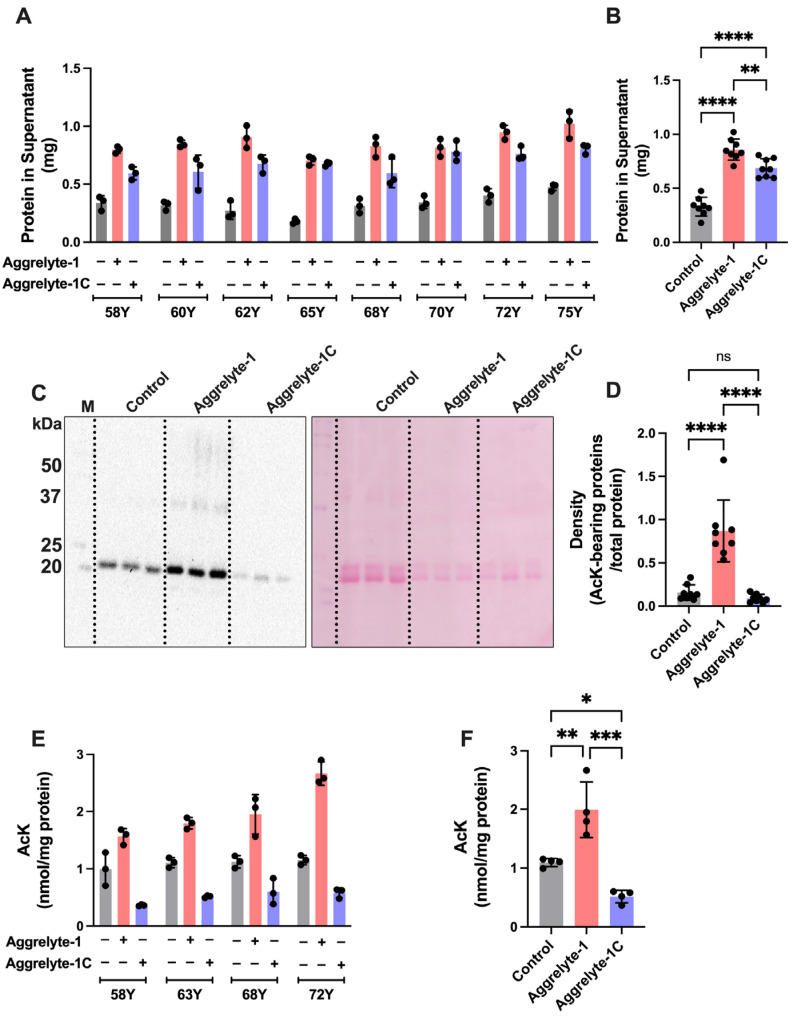
Aggrelytes increased the solubility of human lens WI and increased AcK levels in solubilized proteins. Two milligrams of WI from the human lens (58–75 years) was suspended in 0.4 mL of 50 mM phosphate buffer, pH 7.4, and the solutions were incubated with 2000 µM aggrelyte-1 or aggrelyte-1C for 24 h at 37 °C. Each sample was separately processed three times and analyzed (mean ± S.D.) (**A**). The combined effects of the aggrelytes on the solubilization of WI (from 8 lenses) are shown in panel (**B**). Representative Western blotting image shows the increase in the AcK-bearing protein content after aggrelyte-1 treatment (**C**). The samples were separately processed three times and analyzed. Five micrograms of protein from each sample were used for Western blotting. The ponceau S-stained membrane showed protein loading. WS from 8 lenses (58 to 75 years) was separately processed three times and analyzed by Western blotting for AcK-bearing proteins. The combined values obtained from the protein of 8 lenses (mean ± S.D.) are shown (**D**). WS (from 58-, 63-, 68- and 72-year-old human lenses) samples were digested with a series of enzymes and analyzed by LC–MS/MS for AcK content. Each sample was processed in triplicate and analyzed, and the bar graphs represent the mean ± S.D. (**E**). A combined plot of the mean values of the three analyses of each sample is shown in (**F**). * *p* < 0.05, ** *p* < 0.01, *** *p* < 0.001, **** *p* < 0.0001, ns = not significant.

We used eight lenses (58–75 years) to determine the effect of 2000 µM aggrelytes on solubilizing WI. Figure 3A shows the solubility of WI (2 mg/0.4 mL) after incubation with 2000 µM aggrelytes. The results indicated that aggrelyte-1-treated samples had higher amounts of solubilized protein (0.70–1.02 mg) than the untreated controls (0.18–0.47 mg) and samples treated with aggrelyte-1C (0.59–0.80 mg). The total solubilized protein from eight lenses treated with aggrelyte-1 was 0.86 ± 0.10 mg (mean ± S.D.), which was 2.6-fold higher (*p* < 0.0001) than that of the controls and 1.2-fold higher than that of the aggrelyte-1C-treated samples (*p* < 0.0001, Figure 3B). The soluble protein content was slightly higher in these experiments compared to those observed in the earlier experiments that determined the solubility as a function of aggrelyte concentration (Figure 2A). The use WI from different lenses, although from comparable ages, could have contributed to this discrepancy.

Figure 3C shows a representative Western blotting image (WI from a 60-year-old human lens) for AcK-bearing proteins after treatment with aggrelyte-1 and ponceau-S staining of the membrane. The AcK content in the solubilized proteins of eight lenses is shown in Figure S2. The aggrelyte-1-treated sample (2000 µM) showed significantly higher AcK content than the controls and aggrelyte-1C-treated samples. When the values were combined, the aggrelyte-1 samples showed a 5.5- and 9.8-fold increase (*p* < 0.0001) compared with the controls and aggrelyte-1C-treated samples, respectively (Figure 3D). The AcK levels measured by LC–MS/MS were 1.56–2.67 nmol/mg protein in the aggrelyte-1-treated samples, whereas they were 0.36–0.60 nmol/mg protein and 0.99–1.15 nmol/mg protein in the aggrelyte-1C and control samples, respectively (Figure 3E). The combined mean values were significantly 1.8-fold higher (*p* < 0.01) in the AcK levels in aggrelyte-1 treated samples compared with the controls (Figure 3F). The aggrelyte-1-treated samples had significantly (*p* < 0.001) higher levels of AcK than the aggrelyte-1C-treated samples. Surprisingly, the levels in the aggrelyte-1C-treated samples were significantly (*p* < 0.05) lower than those in the controls; the reason for this is unknown at this time.

### 2.3. Aggrelytes Increase the Protein–Thiol Content

**Figure 4:**
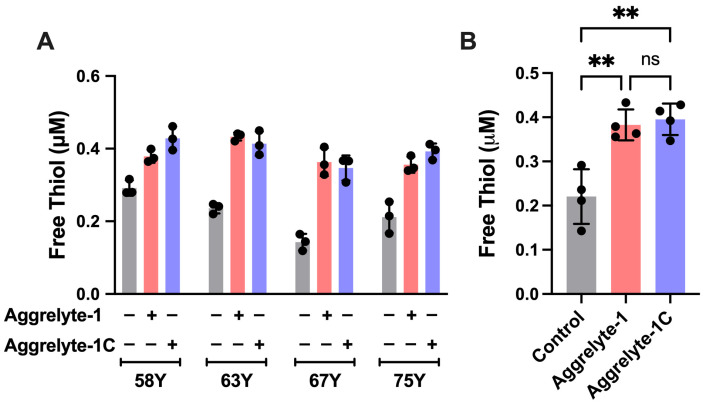
Aggrelytes increased the protein–thiol content in solubilized proteins. The protein–thiol content in the solubilized proteins of aged lenses (58, 63, 67, and 75 years old) was determined using a Thiol Quantification Assay Kit as described in the Methods. The data are shown as the mean ± S.D. for each sample processed three times separately and analyzed (**A**). A combined plot of the mean values of the three analyses of each sample is shown on the right (**B**). ** *p* < 0.01, ns = not significant.

The thiol levels were 0.14–0.29 µM in the control samples but 0.36–0.43 µM and 0.35–0.43 µM in the aggrelyte-1- and aggrelyte-1C-treated samples, respectively (Figure 4). The aggrelyte-1- and aggrelyte-1C-treated samples had similar levels. These results suggested that both aggrelytes reduced protein disulfide bonds with comparable efficiency.

### 2.4. Aggrelyte-1 Mildly Alters the Structure of α-Crystallin

The aggrelyte-1-solubilized proteins contained substantial amounts of αAC and αBC (Figure S3A). Therefore, we investigated the effects of aggrelyte-1 on the structure of these two proteins. The fluorescence spectra of αAC (10 µM) treated with aggrelyte-1 (0–100 µM; Figure S3B) showed a concentration-dependent quenching. The saturation in tryptophan fluorescence with increasing concentrations of aggrelyte-1 provided further evidence for aggrelyte-1 binding to αAC. We did not observe any changes in the fluorescence spectra of αBC (Figure S3C). The far-UV CD results did not show any changes in the secondary structure of the two proteins after aggrelyte-1 treatment (Figure S3D,E). However, the near-UV CD spectra showed mild structural changes (at a 1:6 molar ratio of α-crystallin to aggrelyte-1) around phenylalanine, tyrosine, and tryptophan residues (Figure S3F,G). This suggested alterations in the tertiary structure of αAC and αBC upon treatment with aggrelyte-1. The bis-ANS fluorescence spectra of aggrelyte-1-treated αAC showed an increase in intensity with a hypsochromic shift in the emission maxima compared with the αAC control, but no measurable effect in αBC was found (Figure S3H,I). Upon treatment with aggrelyte-1, the chaperone activity, measured using alcohol dehydrogenases (ADH) as a client protein, improved in both αAC and αBC (Figure S4).

### 2.5. Aggrelytes Are Not Cytotoxic

Before determining the effect of aggrelytes on lens stiffness ex vivo, we determined whether aggrelytes had cytotoxic effects on mouse and human lens epithelial cells. Our results suggested no cytotoxicity up to 2000 µM of aggrelytes in mouse and human lens epithelial cells after 24 or 72 h of treatment (Figure S5). Human (age: 44–65 years) and mouse (C57BL/6J, age: 12 months) lenses were treated with aggrelyte-1 or aggrelyte-1C for 24 and 72 h, respectively. We did not observe significant changes in either lens transparency (mouse and human) or weight (human) after aggrelyte treatment (Figures S6 and S7). Together, these results suggested that under our experimental conditions, aggrelytes were not cytotoxic and did not reduce lens transparency.

### 2.6. Aggrelytes Reduce Lens Stiffness

**Figure 5:**
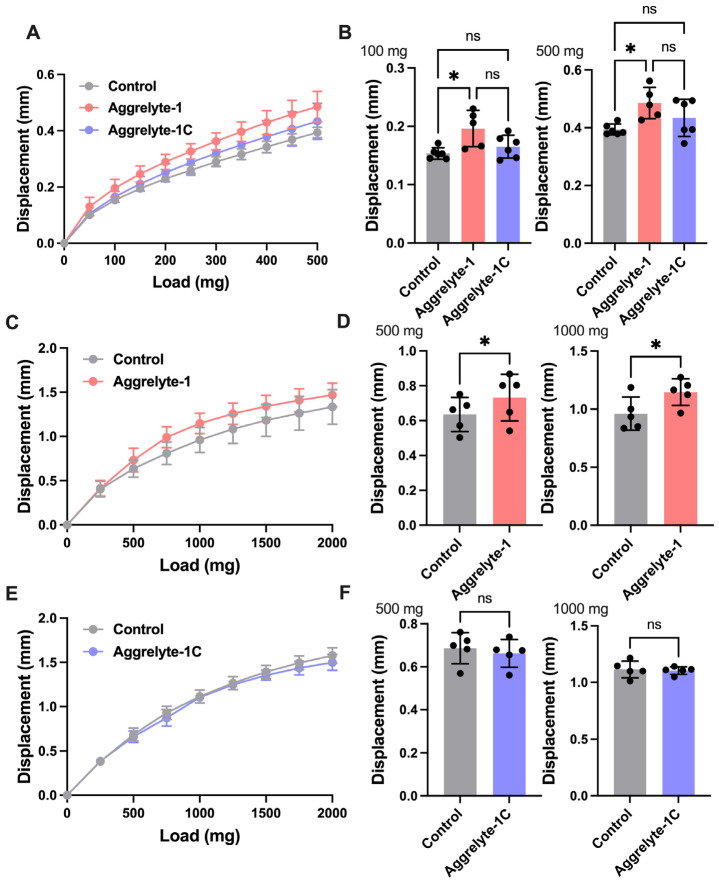
Aggrelyte-1 decreased the stiffness of mouse and human lenses. Mouse lenses (from 7-month-old C57BL6/J mice) and human lenses (44–66 years) cultured ex vivo were treated with 2000 μM aggrelyte-1 or aggrelyte-1C for 24 h and 72 h, respectively, in serum-free MEM as described in the Methods section. Lens stiffness was measured using a computer-controlled lens squeezer (**A**,**C**,**E**). The lens displacement was measured at various loads. The bar graphs on the right show displacement at 100 mg and 500 mg load for mouse lenses (**B**) and 500 mg and 1000 mg load for human lenses (**D**,**F**). The bar graphs represent the mean ± S.D. of 5–6 measurements. * *p* < 0.05, ns = not significant.

Figure 5A shows the load versus displacement plot for mouse lenses (age: 7 months) in culture after treatment with 2000 µM aggrelyte-1 or aggrelyte-1C for 24 h. Aggrelyte-1-treated lenses showed a significant (*p* < 0.05) increase in displacement at 100 and 500 mg loads over controls (Figure 5B), suggesting a decrease in lens stiffness. The displacement was slightly higher in the aggrelyte-1C treated lenses but not significantly different than those in the controls or aggrelyte-1 treated lenses. Human lenses (age: 44–66 years) were treated with 2000 µM aggrelyte-1 or aggrelyte-1C for 72 h, and the medium was replaced with fresh aggrelytes every 24 h. These lenses exhibited significant displacement increases over controls after treatment with aggrelyte-1 at 500 and 1000 mg loads (Figure 5C,D) (*p* < 0.05). However, the aggrelyte-1C-treated human lenses did not show significant changes in displacement compared with the controls at equal loads (Figure 5E,F). Our results suggested that aggrelyte-1, but not aggrelyte-1C treatment reduced lens stiffness compared to controls (Figure 5 and Figure S8).

**Figure 6:**
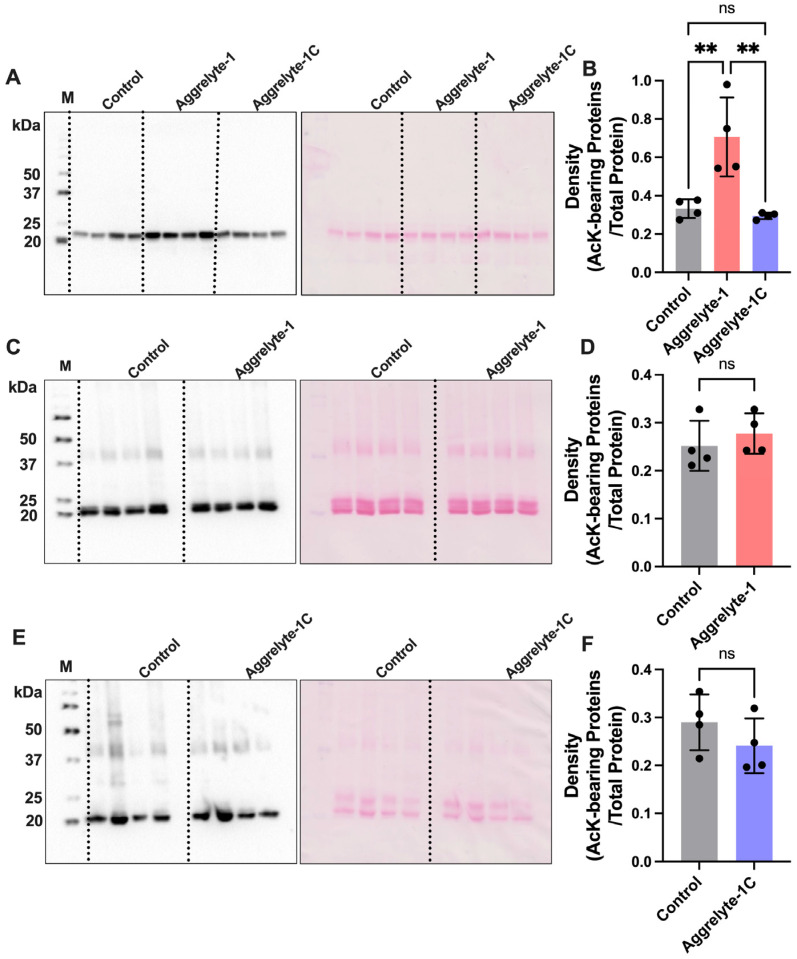
WS from aggrelyte-1 treated lenses contained higher levels of AcK. The levels of AcK-bearing proteins in WS from mouse (**A**,**B**) and human lenses treated with aggrelyte-1 (**C**,**D**) or aggrelyte-1C (**E**,**F**) were determined by Western blot analysis. Twenty micrograms of protein from each sample were used for Western blotting. Ponceau S-stained membranes showed protein loading. Densitometric plots represent the mean ± S.D. of n = 4 measurements. M = molecular weight markers. ** *p* < 0.01, ns = not significant.

After measuring the stiffness, we determined the AcK levels in the WS of lenses. The aggrelyte-1-treated mouse lenses showed significantly higher AcK-bearing protein levels than the aggrelyte-1C-treated (*p* < 0.05) and control lenses (*p* < 0.01, Figure 6A,B). The AcK-bearing protein levels were similar between the aggrelyte-1C treated lenses and controls. In the case of human lenses, aggrelyte-1 increased the AcK level by 6.7% with respect to the controls, but the difference was not significant (Figure 6C,D). Aggrelyte-1C showed an insignificant decrease in the AcK content relative to the controls (Figure 6E,F). Together, these data suggested that aggrelyte-1 permeated lenses, acetylated lysine residues, and reduced stiffness.

**Figure 7:**
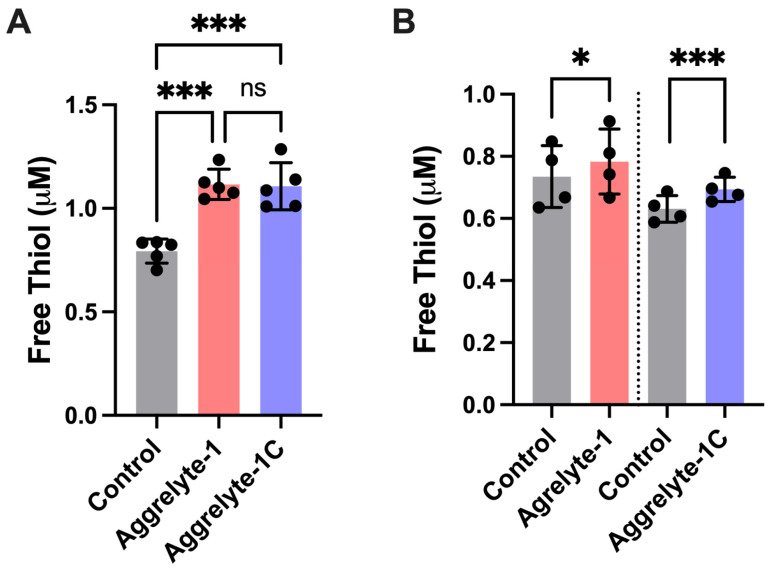
Aggrelytes reduced disulfides in human and mouse lenses. The protein–thiol content in WS (10 µg) of mouse (**A**) and human lenses (**B**) after treatment with aggrelytes (2000 µM, 24 h for mouse and 72 h for human lenses) was measured using the Thiol Quantification Assay Kit as described in the Methods section. * *p* < 0.05, *** *p* < 0.001, ns = not significant.

The free thiol content was measured in the WS of mouse and human lenses. Both aggrelyte-1- and aggrelyte-1C-treated mouse lenses showed a significant ~40% (*p* < 0.001) increase over the controls (Figure 7A). However, the effect in human lenses was modest; aggrelyte-1 and aggrelyte-1C increased the free thiol content by 6.5 and 9.8%, respectively (Figure 7B).

## 3. Discussion

The primary goal of this study was to develop small molecules for reversing the age-associated lens stiffness that contributes to presbyopia. We reasoned that compounds that can solubilize aggregated proteins would reduce the stiffness of aged human lenses, as protein aggregation and insolubilization have been considered primary reasons for the age-associated stiffening of lenses. Our study showed that aggrelyte-1 partially solubilized WI in aged human lenses. The Western blotting and LC–MS/MS data confirmed significant lysine acetylation in the solubilized protein upon aggrelyte-1 treatment. Our study also showed that aggrelyte-1 reduced disulfides in proteins. The ability of aggrelyte-1 to reduce disulfides in proteins must have occurred due to the donation of an acetyl group from the S-acetyl group to lysine residues in proteins and consequent exposure of the -SH group. This dual property could have contributed to the solubilization of human lens WI. This assertion is supported by the data from aggrelyte-1C (which lacked the S-acetyl group); the protein-solubilizing capacity of aggrelyte-1C was significantly lower than that of aggrelyte-1; the free-SH group in the structure likely contributed to its ability to solubilize proteins, but its protein-solubilizing capacity was much lower than that of aggrelyte-1. More importantly, our study showed that aggrelyte-1 could reduce the stiffness of aged mouse and human lenses, and this reduction was accompanied by increased protein thiol and AcK levels.

Previous studies have shown that acetylation enhances side chain size, neutralizes the positive charge of lysine residues, and alters protein properties by increasing hydrophobicity [17,25,26]. We observed that the hydrophobicity of αAC but not αBC increased upon treatment with aggrelyte-1. While the increased hydrophobicity of αAC might make it a better chaperone, it is unlikely to contribute to the aggrelyte-1-mediated solubility of α-crystallins. Interestingly, we found that aggrelyte treatment improved the chaperone activity of both αAC and αBC, which could have played a role in the aggrelyte-mediated solubilization of WI. αAC contains seven lysine residues and has a net surface charge of -16.84 ± 0.64 mV at physiological pH [27]. Acetylation of lysine residues could have increased the net negative charge in αAC, resulting in higher electrostatic repulsions between subunits and between αAC and other proteins, which might have contributed to higher protein solubility. The pI of αAC and αBC is between 4.0 and 6.0 [28]. Therefore, their surface charges are expected to be negative at physiological pH. Acetylation of lysine residues by aggrelyte-1 could have neutralized the surface positive charge and increased the net negative charge, resulting in charge–charge separation of proteins, which could have contributed to protein solubilization. Furthermore, CD and fluorescence spectroscopy revealed that aggrelyte-1 altered the tertiary structure of αAC and αBC, similar to our previous observations [20,24]. The aggrelyte-1-modified αAC and αBC may have adopted conformations in which negatively charged residues were more surface exposed and promoted protein solubility.

The mammalian lens contains high levels of glutathione (GSH) [29]. With aging, the lens loses GSH, which leads to oxidative damage and disulfide-linked protein aggregates [14,29]. A strategy to enhance GSH in the lens to limit oxidative damage and thereby reduce stiffness is feasible but may require regular replenishment of GSH. Lipoic acid choline ester has shown promising results in lowering mouse and human lens stiffness [14,15], works similarly to GSH, and may therefore require periodic replenishments in the lens to maintain sustained effects. Aggrelyte-1, on the other hand, may require less frequent applications, as it acetylates proteins and reduces protein disulfides. As the deacetylation of proteins may not occur in the inner regions of the lens because of negligible sirtuin activity (due to structural modifications of sirtuins or unavailability of the cofactor NAD), the effects of aggrelyte-1 could be long-lasting or permanent.

Our results showed that treatment with aggrelyte-1 decreased the stiffness of aged mouse and human lenses without altering lens transparency or weight. In these lenses, we observed higher thiol and AcK contents, implying that aggrelyte-1 treatment decreased lens stiffness through acetylation followed by disulfide reduction. These observations are significant in the context of reversing presbyopia. We recognize that the aggrelyte-1 concentrations used in this study (up to 2000 µM), although not cytotoxic to lens epithelial cells, may not be ideal for topical applications. At high concentrations, aggrelyte-1 may acetylate proteins in other eye tissues, such as the cornea, ciliary body, and retina. To avoid off-target effects, the cell penetration property of aggrelyte-1 could be improved, which may result in less aggrelyte-1 being required to target lens proteins specifically. Other strategies could include linking or encapsulating aggrelyte-1 in a carrier and intracameral injection such that aggrelyte-1 is delivered proximal to the lens for more specific uptake by the lens. Furthermore, additional work is required to understand the specific effects of aggrelyte-1 on lens cytosolic, cytoskeletal, and membrane proteins. Given these limitations, the results of this study should be treated as a proof of concept. Further refinement of the structure of aggrelyte-1 for better penetration and stability and the development of methods to deliver it into the lens could lead to novel methods for reversing presbyopia.

## 4. Materials and Methods

### 4.1. Chemicals

*S*-Acetyl glutathione (Product# AMBH95E07091) and alcohol dehydrogenase (ADH) (Product# A7011) were obtained from Sigma Aldrich (St. Louis, MO, USA). Monoclonal antibodies against acetyllysine (AcK) (Cat# 9681S) and horseradish peroxidase (HRP)-conjugated horse anti-mouse IgG (Cat# 7076S) were purchased from Cell Signaling Technology (Danvers, MA, USA). All other chemicals were of analytical grade.

### 4.2. Synthesis of Aggrelyte-1

**Scheme 1:**
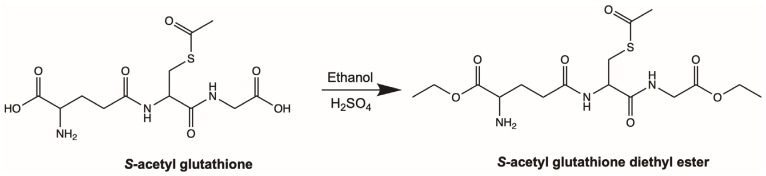
The synthesis procedure for S-acetyl glutathione diethyl ester.

*S*,*N*-diacetyl glutathione diethyl ester was synthesized in a two-step process. In the first step (Scheme 1), sulfuric acid (2 mL) was added to a slurry of *S*-acetyl glutathione (1 g) in anhydrous ethanol (20 mL) at 0 °C until a clear solution was obtained. The reaction mixture was stirred at 4 °C for 7 days. The solvent was removed under vacuum and the gummy residue was dissolved in DMSO and subjected to preparative HPLC [column: Biotage Sfär C18 Duo, 100 Å 30 μm (Biotage, Uppsala, Sweden), solvent A: water +0.1% TFA and solvent B: 95% acetonitrile in water +0.1% TFA, 10–95% gradient of B; 0–10 min: 10% B, 10–25 min: 10–100% B, flow rate 15 mL/min]. The eluant was monitored with an online UV detector set at 215 nm. The product eluted at an R_t_ of 16.2 min was collected and lyophilized. The resulting glassy powder was stored after nitrogen flushing and vacuum sealing (yield, 27%). ^1^H NMR (400 MHz, CDCl_3_); δ 1.25 (t, 6H), 2.38 (m, 2H), 2.34 (s, 3H), 2.62 (m, 2H), 3.17 (m, 1H), 3.30 (m, 1H), 3.97 (d, 2H), 4.17 (m, 3H), 4.26 (q, 2H), 4.67 (m, 1H), 6.9 (bs, 2H), 7.49 (s, 1H), 7.93 (s, 1H). ESI-MS, calculated m/z = 405.15, found m/z = 405.5 [M+], 407.7 [M + 2].

**Scheme 2:**
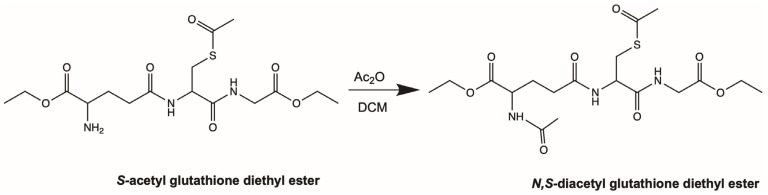
The synthesis procedure for *N*,*S*-diacetyl glutathione diethyl ester.

In the second step (Scheme 2), diisopropyl ethyl amine (113 μL, 0.65 mmol) was added to an ice-cooled suspension of *S*-acetyl glutathione diethyl ester (66 mg, 0.16 mmol) in anhydrous dichloromethane (DCM, 10 mL) followed by acetic anhydride (Ac_2_O, 46 μL, 0.49 mmol). The resulting clear solution was stirred at 0 °C for 10 min and then at room temperature for 2 h. The solvent was removed under a vacuum, and the gummy residue was dissolved in DMSO and subjected to preparative HPLC (column: Biotage Sfär C18 Duo, 100 Å 30 μm, solvent A: water +0.1% TFA and solvent B: 95% acetonitrile in water +0.1% TFA, 10–95% of gradient B; 0–6 min: 10% B, 6–16 min: 10–100% B, flow rate 15 mL/min). The eluant was monitored with an online UV detector set at 215 nm. The product eluted at an R_t_ of 11.5 min was collected and lyophilized. The glassy powder obtained was stored after nitrogen flushing and vacuum sealing (yield, 63%). ^1^H NMR (400 MHz, CDCl_3_); δ 7.09 (bs, 1H, NH), 6.81 (m, 1H, NH), 6.51 (m, 1H, NH), 4.64 (m, 2H, CH_2_), 4.21 (m, 4H, OCH_2_), 4.08 (dd, *J* = 18.2, 5.8 Hz, 1H, CH_2_), 3.95 (dd, *J* = 18.1, 5.1 Hz, 1H, CH_2_), 3.38 (dd, *J* = 14.4, 4.5 Hz, 1H, CH_2_), 3.26 (dd, *J* = 14.4, 8.1 Hz, 1H, CH_2_), 2.37 (s, 3H, CH_3_), 2.32 (m, 2H, CH_2_), 2.22 (m, 1H, CH), 2.03 (s, 3H, CH_3_), 2.00 (m, 1H, CH), 1.27 (m, 6H, CH_3_).

### 4.3. Synthesis of Aggrelyte-1C

**Scheme 3:**
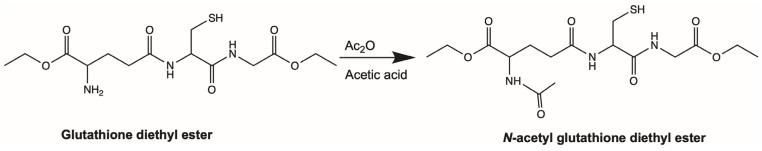
The synthesis procedure for N-acetyl glutathione diethyl ester.

To an ice-cold suspension of glutathione diethyl ester (100 mg, 0.27 mmol) in water (5 mL), a mixture of Ac_2_O (2 mL) and acetic acid (2 mL) was added over 1 h (Scheme 3). The mixture was chilled on ice. Additional acetic anhydride (2 mL) was added, and the reaction mixture was stirred while it gradually warmed to room temperature overnight. The solvent was removed under a vacuum, and the gummy residue was dissolved in DMSO and subjected to preparative HPLC (column: Biotage Sfär C18 Duo, 100 Å 30 μm, solvent A: water +0.1% TFA and solvent B: 95% acetonitrile in water +0.1% TFA, 10–95% of gradient B; 0–7 min: 10% B, 7–17 min: 10–100% B, flow rate 15 mL/min). The eluant was monitored with an online UV detector at a wavelength of 215 nm. The product eluted at an R_t_ of 10.7 min was collected and lyophilized. The powder obtained was stored in vacuum under nitrogen (yield, 46%). ^1^H NMR (400 MHz, D_2_O); δ 4.57 (m, 1H, CH), 4.36 (m, 1H, CH), 4.22 (m, 4H, OCH_2_), 4.03 (d, *J* = 3.8 Hz, 2H, CH_2_), 2.93 (t, *J* = 5.9 Hz, 2H, CH_2_), 2.48 (t, *J* = 7.6 Hz, 2H, CH_2_), 2.21 (m, 1H, CH), 2.05 (s, 3H, COCH_3_), 2.00 (m, 1H, CH), 1.27 (m, 6H, CH_3_).

### 4.4. Isolation of Water-Insoluble (WI) Protein from Aged Human Lenses

Human lenses (donor age: 58–75 years) were obtained from Saving Sight, Kansas City, MO, and Lions Eye Institute for Transplant & Research, Tampa, FL, on dry ice. The lenses were harvested within 36 h postmortem and stored at −80 °C until use. Each lens was thawed on ice, decapsulated, and homogenized in a hand-held glass homogenizer in 1.5 mL of N_2_-bubbled PBS. The homogenate was centrifuged at 20,000× *g* for 20 min at 4 °C. The resulting pellet (WI) was washed with 1 mL of N_2_ bubbled PBS and lyophilized.

### 4.5. Protein Solubility by Aggrelytes

Stock solutions (20 mM) of aggrelytes were prepared in 50 mM phosphate buffer, pH 7.4. Two milligrams of WI were suspended in 0.4 mL of 50 mM phosphate buffer, pH 7.4, containing 0.002% sodium azide. Aggrelyte-1 or aggrelyte-1C was added to the protein suspension to attain a final concentration of 0–2000 µM. The mixture was incubated in a shaker for 24 h at 37 °C. The suspension was centrifuged at 20,000× *g* for 20 min at 4 °C. The resulting supernatant was collected, and its protein content was measured using the BCA Protein Assay Kit from Thermo Scientific (Waltham, MA, USA) using BSA as the standard. Different concentrations of aggrelyte-1 and aggrelyte-1C (0–2000 µM) were used in the BCA assay for background correction.

### 4.6. Western Blotting for AcK and Crystallin Subunits

The solubilized proteins were subjected to SDS–PAGE on a 12% gel. The proteins were electrophoretically transferred to a nitrocellulose membrane. The membrane was blocked with 5% nonfat dry milk for 1 h and incubated with the AcK antibody (dilution: 1:5000), followed by HRP-conjugated horse anti-mouse IgG (dilution: 1:5000). The membrane was developed using the Enhanced Chemiluminescence Detection Kit (Thermo Scientific) and imaged in the Chemidoc System (Bio-Rad, Hercules, CA, USA). Ponceau-S staining was used to visualize protein loading levels.

Western blotting of the solubilized protein of human lenses for α-crystallin subunits (αAC and αBC) was performed using specific primary antibodies against αA-crystallin (αAC, Enzo Life Sciences, Cat# ADI-SPA-221D, dilution: 1:14,000) and αB-crystallin (αBC, from The University of Iowa, dilution: 1:700,000). HRP-conjugated goat anti-rabbit IgG (Cat# 7074S, dilution: 1:5000) was used as the secondary antibody for αAC, and HRP-conjugated goat anti-mouse IgG (dilution: 1:5000) was used for αBC.

### 4.7. LC–MS/MS Measurement of AcK

We used a series of enzymes to digest the solubilized protein (0.90 mg/mL) as previously described [21]. The standard addition method was used to quantify AcK by LC–MS/MS, as previously described [21].

### 4.8. Free Protein–Thiol Estimation

After 24 h of incubation of WI with aggrelytes and centrifugation, the resulting soluble protein, and WS isolated from human and mouse lenses treated with aggrelytes (see below) were immediately filtered through a 10 kDa cutoff centrifugal filter at 4 °C. Filtration was repeated four times by adding 0.45 mL of N_2_-bubbled 50 mM sodium phosphate buffer, pH 7.4 to the retentate. Protein was measured by the BCA assay, as described above. Ten micrograms of protein were used for thiol content determination using the Thiol Quantification Assay Kit (Abcam, Cambridge, MA, USA), and reduced GSH served as the standard.

### 4.9. Fluorescence and CD Experiments

Fluorescence spectra were measured using a Fluoromax-4 spectrofluorometer (Horiba Jobin Yvon, Edison, NJ, USA). Tryptophan fluorescence was recorded at an excitation wavelength of 295 nm; the emission spectra were recorded from 310 nm to 470 nm at 2 nm intervals using 5 nm slit widths.

Far and near-UV CD spectra were reordered at a protein concentration of 0.24 mg/mL in a 0.1 cm pathlength cuvette and 1 cm pathlength cuvette, respectively, in a Chirascan Plus spectrophotometer (Applied Photophysics, Leatherhead, Surrey, UK). Data were collected at a scanning speed of 1 nm/s at a 1 nm bandwidth in the 200–250 nm wavelength range for far-UV CD and 250–350 nm for near-UV CD. The buffer spectra were subtracted, and the final spectra were generated after averaging three successive measurements.

Bis-ANS binding assays were performed to determine the effects of aggrelyte-1 treatment on the surface hydrophobicity of αAC and αBC. Aggrelyte-1-treated or untreated α-crystallin (20 µM) was incubated with bis-ANS (20 µM) for 2 h in 50 mM sodium phosphate buffer, pH 7.4. The fluorescence of the samples was recorded from 390 nm to 700 nm (excitation = 370 nm).

### 4.10. Chaperone Activity Assay

αAC or αBC (4 mg/mL) was incubated with 2000 µM aggrelyte-1 at 37 °C for 16 h in 50 mM sodium phosphate buffer, pH 7.4. For the chaperone assay, 0.3 mg/mL ADH was added to 6.0 µg/mL αAC or αBC in 50 mM phosphate buffer, 7.0 (total assay volume = 200 µL) and incubated at 49 °C over a 2 h period. Light scattering at 360 nm was monitored using a microplate reader (Spectramax ID3, Molecular Devices, Sunnyvale, CA, USA).

### 4.11. Toxicity Studies

Mouse lens epithelial cells (primary cells from lenses of 4- to 6-week-old C57BL/6J mice, passages 3–5) and human lens epithelial cells (primary cells from 41-year-old noncataractous lens, passages 3–5) were incubated with varying concentrations of aggrelyte-1 or aggrelyte-1C (from 0 to 2000 µM) for 24 h and 72 h. The medium containing freshly dissolved aggrelytes was changed for human lens epithelial cells every 24 h. An MTT assay was used to determine cell viability.

### 4.12. Lens Stiffness Measurements

All animal experiments were reviewed and approved by the Institutional Animal Care and Use Committee (IACUC) of the University of Colorado, Aurora, and performed in adherence to the ARVO Statement for the Use of Animals in Ophthalmic and Vision Research. Seven-month-old mice (C57BL/6J) and human lenses (age: 44–66 years) were used for the study. Freshly dissected mouse lenses (by a posterior approach) were incubated in serum-free and phenol red-free MEM at 37 °C for 2 h, after which the medium was changed to MEM containing aggrelyte-1 or aggrelyte-1C and incubated for 24 h at 37 °C. Human lenses were incubated in phenol red-free MEM with or without aggrelyte-1 (2000 µM) for 72 h, and the medium containing aggrelytes was changed every 24 h. The absence of aggrelyte-1 or aggrelyte-1C was used as a control. Lens stiffnesses were measured using a fully automated squeezing system described elsewhere [30]. A computer-controlled linear actuator (LTA-HS Precision Motorized Actuator, Newport) was mounted above the lenses to apply compressive force through a load cell. Two CCD side cameras were used to check the lens orientation. A force step method was applied with ten successive 50 (mouse) or 250 (human) mg incremental loads followed by unloading steps of 20 s. MATLAB (R2007a, Mathworks) written software was used to control the system. The vertical movement of the actuator was driven by a Motion Controller/Driver (SMS-100 series, NEWPORT Inc.), and the output data were acquired at 5 Hz via the RS-232 port. The generated data were analyzed using Origin 2021b Software (OriginLab Cor., Northampton, MA, USA). A changepoint analysis of load versus displacement was performed. After measuring stiffness, mouse lenses were homogenized in 0.2 mL of 50 mM phosphate buffer, pH 7.4. Human lenses were decapsulated and homogenized in 1.5 mL of 50 mM phosphate buffer, pH 7.4 and centrifuged at 20,000× *g* for 20 min at 4 °C to obtain the WS fraction.

### 4.13. Statistics

One-way ANOVA and Tukey’s multiple comparison test were used to analyze the statistical data using GraphPad Prism (GraphPad Software, La Jolla, CA, USA). All experiments had at least three independent analyses (n ≥ 3), and all data are presented as the mean ± standard deviation (SD).

## Data Availability

The authors confirm that the data supporting the findings of this study are available within the article and its Supplementary Materials.

## Supplementary Materials

The following supporting information can be downloaded at: https://www.mdpi.com/article/10.3390/ijms24032196/s1.

## Abbreviations

WI: water-insoluble protein; WS, water-soluble protein; αAC, αA-crystallin; αBC, αB-crystallin; AcK, *N^e^*-acetyllysine; ADH, alcohol dehydrogenase.
