# Supplementary material for: Promotion of Protein Solubility and Reduction in Stiffness in Human Lenses by Aggrelyte-1: Implications for Reversing Presbyopia
Source: Int J Mol Sci. 2023 Jan 22;24(3):2196. doi: 10.3390/ijms24032196 (PMC9917358; doi:10.3390/ijms24032196)
Supplement: Supplementary file 1 [file ijms-24-02196-s001.zip › ijms-2126010-supplementary.pdf]

Figure S1

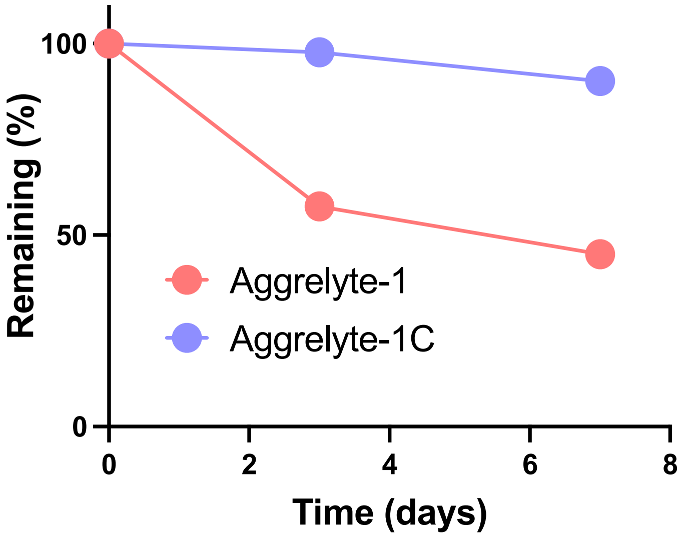

Figure S1. Stability of aggrelyte-1 and 1C. Aggrelytes (each 2 mg/mL) were incubated in 50 mM phosphate buffer, pH 7.4, for up to 7 days at 37 °C. Aliquots were withdrawn at different time points. NMR spectroscopy was used to determine their stability.

Figure S2

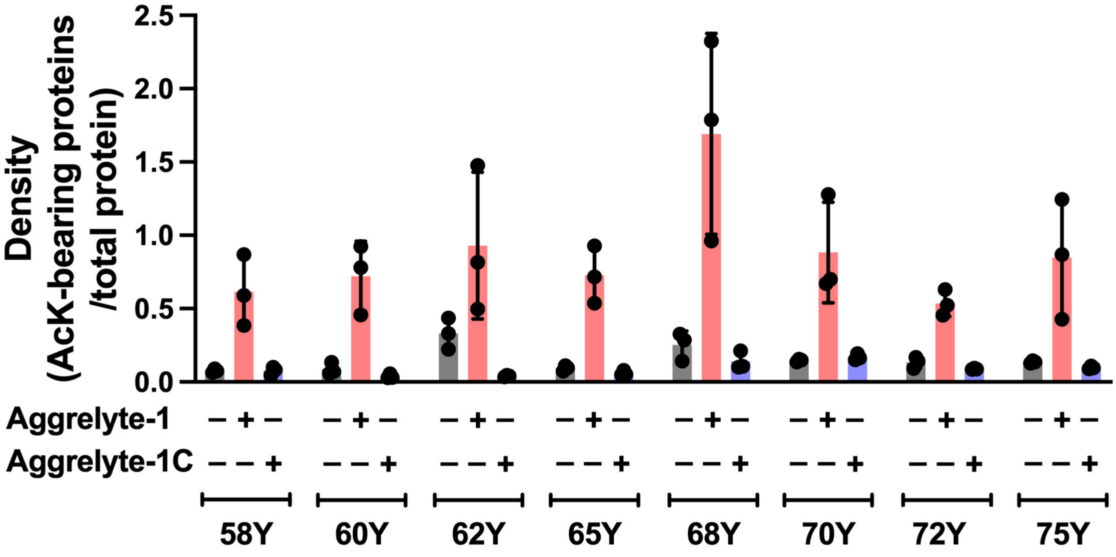

Figure S2. AcK-bearing protein content in aggrelyte-1 solubilized proteins. WI (2 mg/0.4 mL) from lenses (age: 58-75 years) was treated with 2000 µM of aggrelytes for 24 h. WS from lenses was separately processed three times and analyzed by Western blotting for AcK-bearing proteins. The bar graphs represent the mean ± S.D. of triplicate measurements.

Figure S3

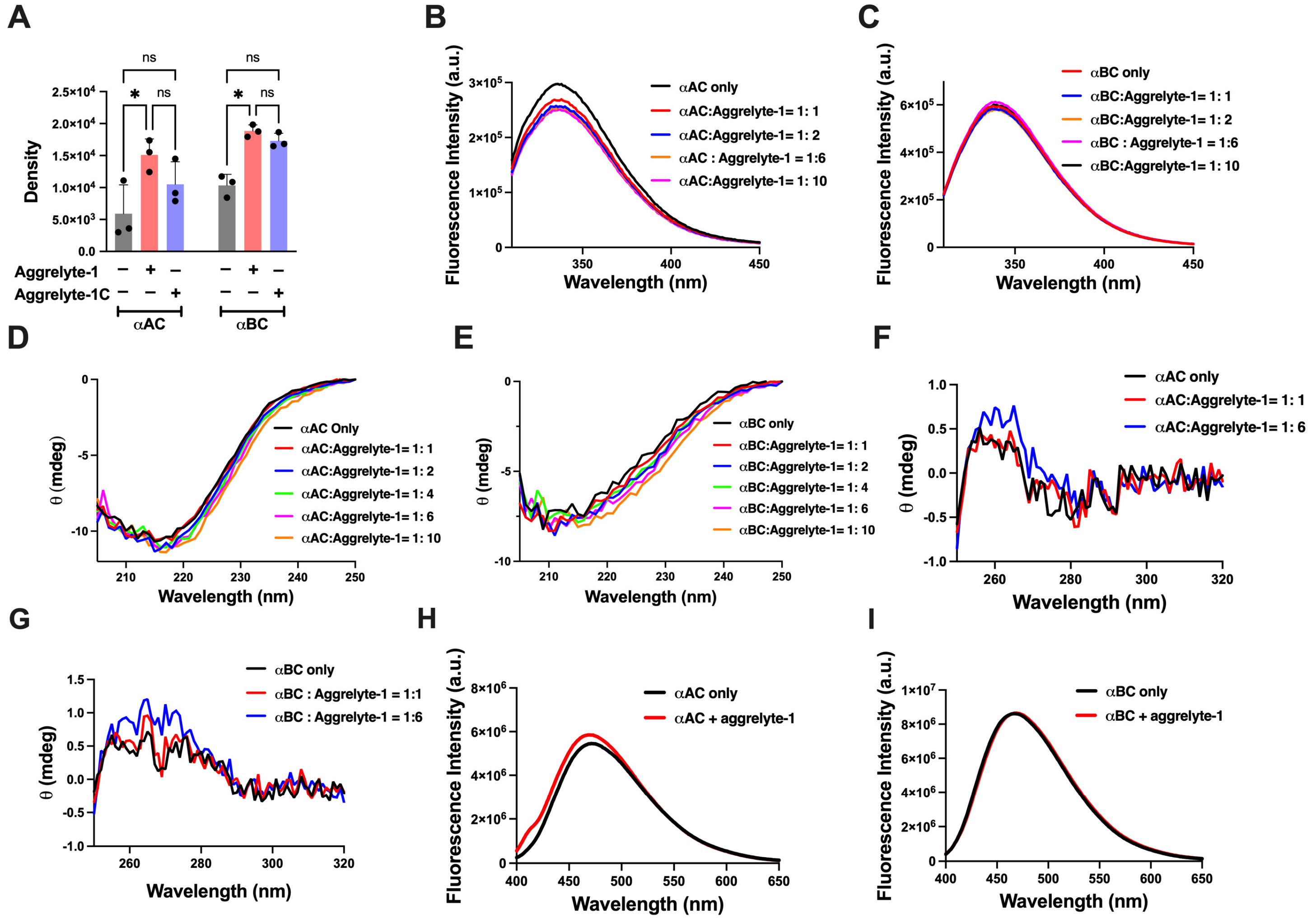
A
Density
ns
*
Aggrelyte-1
Aggrelyte-1C
αAC
αBC
B
Fluorescence Intensity (a.u.)
Wavelength (nm)
αAC only
αAC:Aggrelyte-1= 1: 1
αAC:Aggrelyte-1= 1: 2
αAC : Aggrelyte-1 = 1:6
αAC:Aggrelyte-1= 1: 10
C
αBC only
αBC:Aggrelyte-1= 1: 1
αBC:Aggrelyte-1= 1: 2
αBC : Aggrelyte-1 = 1:6
αBC:Aggrelyte-1= 1: 10
D
θ (mdeg)
αAC Only
αAC:Aggrelyte-1= 1: 1
αAC:Aggrelyte-1= 1: 2
αAC:Aggrelyte-1= 1: 4
αAC:Aggrelyte-1= 1: 6
αAC:Aggrelyte-1= 1: 10
E
αBC only
αBC:Aggrelyte-1= 1: 1
αBC:Aggrelyte-1= 1: 2
αBC:Aggrelyte-1= 1: 4
αBC:Aggrelyte-1= 1: 6
αBC:Aggrelyte-1= 1: 10
F
αAC only
αAC:Aggrelyte-1= 1: 1
αAC:Aggrelyte-1= 1: 6
G
αBC only
αBC : Aggrelyte-1 = 1:1
αBC : Aggrelyte-1 = 1:6
H
αAC only
αAC + aggrelyte-1
I
αBC only
αBC + aggrelyte-1

Figure S3. α-Crystallin levels in solubilized protein and structural changes in αAC and αBC after treatment with aggrelytes. The levels of αAC and αBC were measured by Western blot analysis followed by densitometry in solubilized WI (73-old lens) treated with or without aggrelyte-1 or aggrelyte-1C (2000 μM) **(A)**. Other details are described in Figure 2. The bar graph represents the mean ± S.D. of n=3 measurements. αAC and αBC were treated with aggrelytes in 50 mM phosphate buffer, pH 7.4, for 2 h. The concentrations of aggrelyte-1 varied from 0 to 10 molar excess of αAC and αBC (10 μM). Tryptophan fluorescence **(B and C),** near-UV CD **(D and E)** and Far UV CD spectra **(F and G)** of aggrelyte-treated samples are shown. Aggrelyte-1 treated and untreated αAC and αBC (20 μM) were incubated with ANS (20 μM), and the fluorescence spectra were recorded at an excitation wavelength of 370 nm **(H and I)**. *$p<0.05$, ns=not significant.

Figure S4

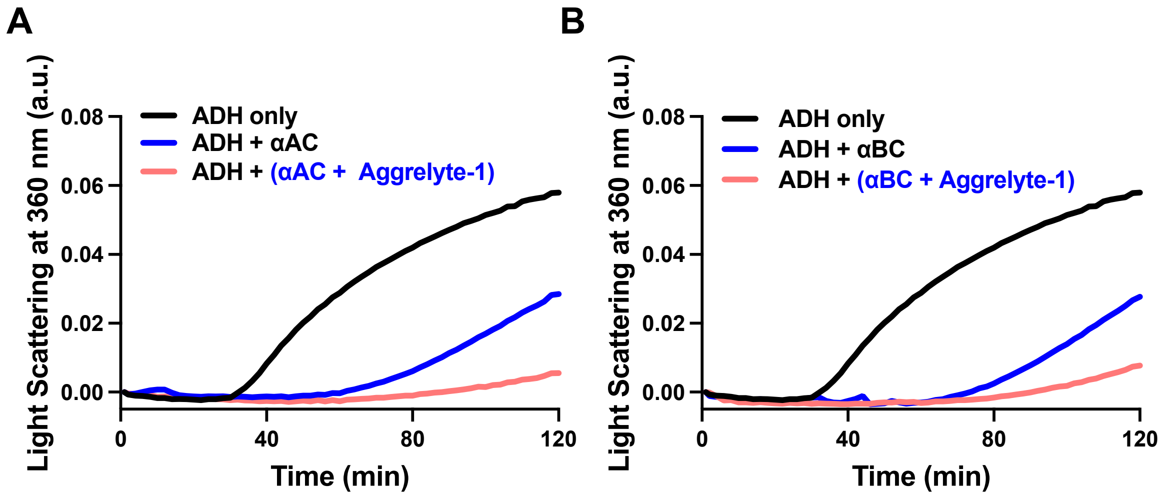

Figure S4. Aggrelyte-1 treatment increases the chaperone activity of crystallins. The chaperone activity of αAC (**A**) or αBC (**B**), at 6.0 μg/mL, treated with or without aggrelyte-1 (16 h in 50 mM sodium phosphate buffer, pH 7.4, at 37 °C) was measured by the thermal aggregation of ADH (0.3 mg/mL) at 49 °C in 50 mM phosphate buffer, pH 7.0 (total volume of the assay = 200 μl).

Figure S5

A

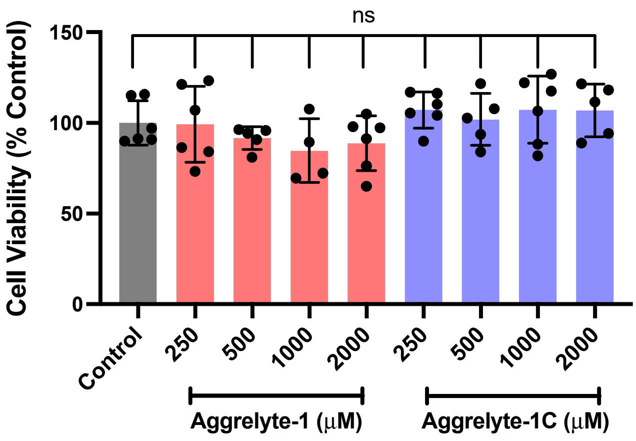

B

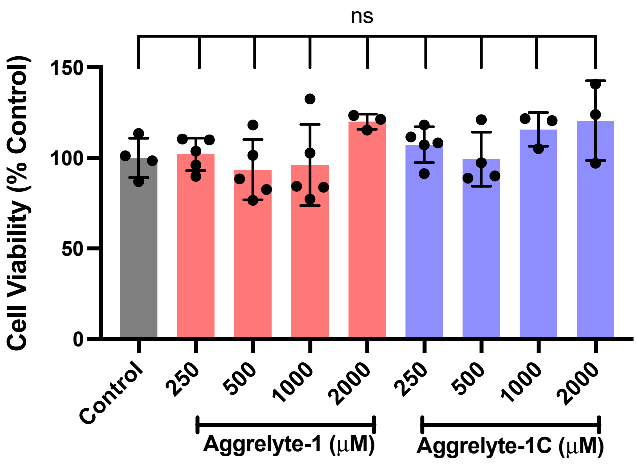

Figure S5. Effects of aggrelytes on lens epithelial cell viability. Primary mouse (isolated from 4-6-weeks-old C57BL/6J mice, passages 3-5) **(A)** and human lens epithelial cells (isolated from a 41-year-old noncataractous lens, passages 3-5) **(B)** were incubated with 2000 μM aggrelytes for 24 h mouse) and 72 h (human, with a change in media containing freshly dissolved aggrelytes every 24 h), respectively. Cell viability was tested by the MTT assay. ns= not significant.

Figure S6

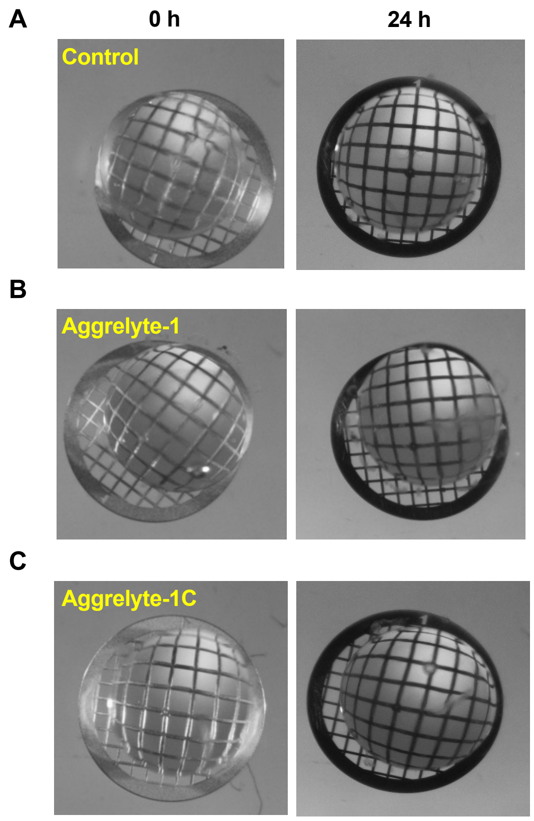

Figure S6. Aggrelytes do not change the transparency of mouse lenses. Mouse lenses (age: 12 months) were incubated without (control) or with aggrelyte-1 or aggrelyte-1C (2000 µM) in serum-free MEM for 24 h as described in the Methods. Representative images show transparency of the control (**A,** n = 2) and aggrelyte-1 (**B,** n = 2) or aggrelyte-1C (**C,** n = 2) treated lenses.

Figure S7

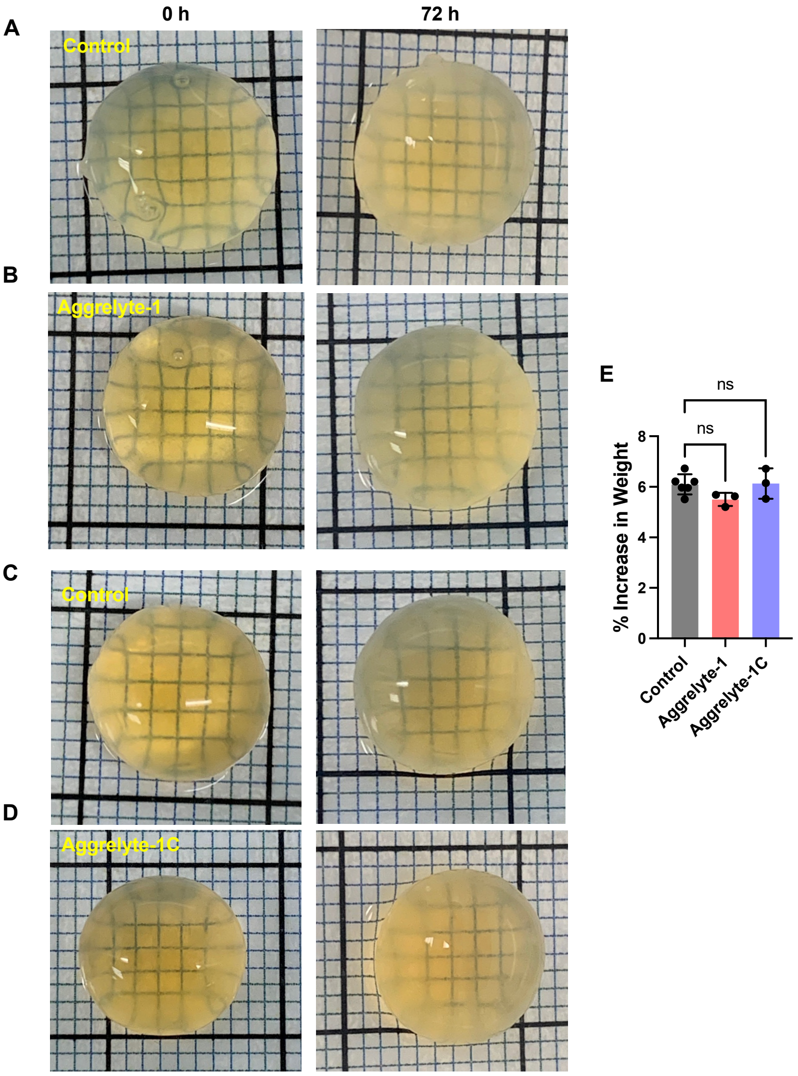

Figure S7. Transparency and weight of human lenses before and after aggrelyte treatment. Representative images of two sets (set 1=A and B, 44 years, and set 2=C and D, 65 years) are shown. Lenses were incubated without (control) or with aggrelyte-1 or aggrelyte-1C (2000 µM) in serum-free MEM for 72 h as described in Methods. The representative images show the transparency of the control (**A** and **C,** n = 3) and aggrelyte-1 (**B,** n = 3) or aggrelyte-1C (**D,** n = 3) treated lenses. The weight of the lenses was measured before and after incubation, and the percentage change in weight was calculated (**E**). ns = not significant.

Figure S8

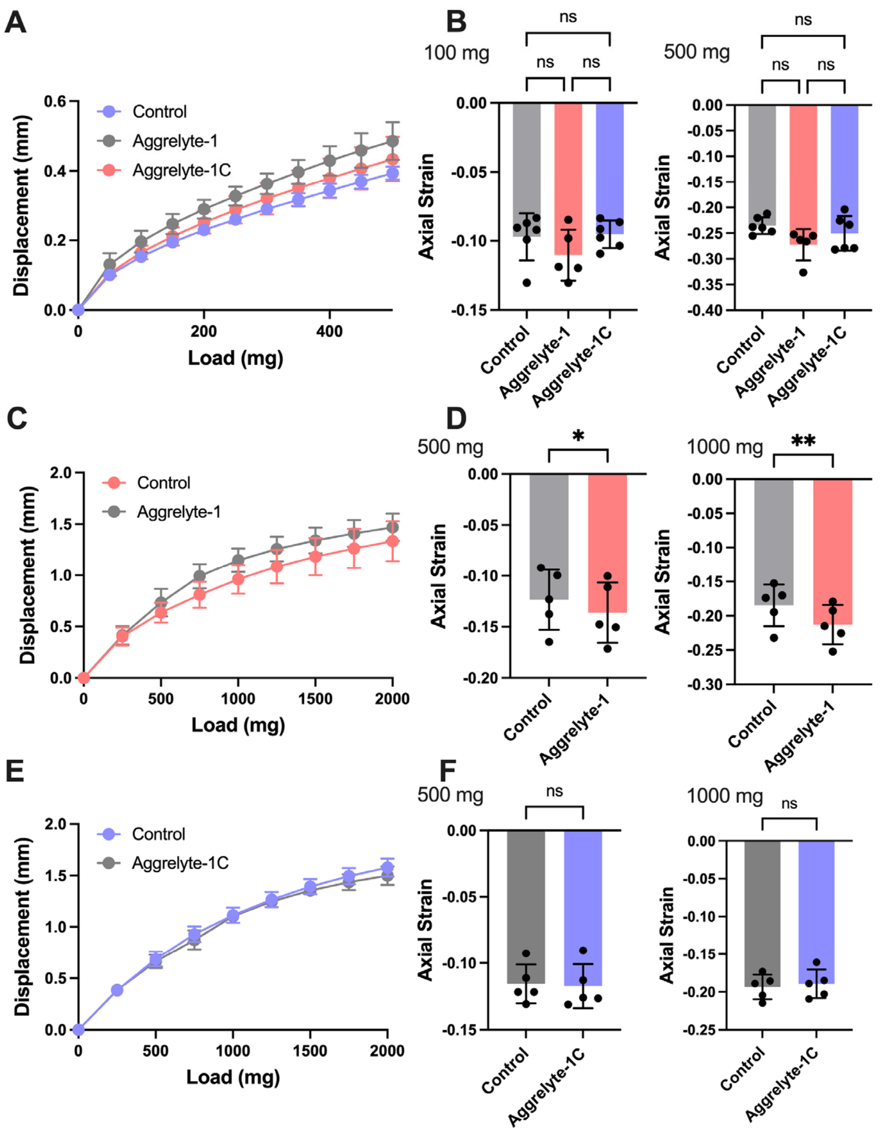

Figure S8. Aggrelyte-1 decreased the axial strain of mouse and human lenses. Mouse lenses (from 7-month-old C57BL6/J mice) and human lenses (44-66 years) cultured *ex vivo* were treated with 2000 µM aggrelyte-1 or aggrelyte-1C for 24 h and 72 h, respectively, in serum-free MEM as described in Figure 5 and the Methods. Lens stiffness was measured using a computer-controlled lens squeezer (A, C and E). The lens displacement was measured at various loads and the axial strain of lenses was calculated based on the following formula:

$$\frac{(\text{diameter of the lens after the load applied-initial diameter of lens})}{\text{The initial diameter of the lens.}}$$

The bar graph on the right shows axial strain at 100 mg and 500 mg load for mouse lenses (B) and 500 mg and 1000 mg load for human lenses (D and F). The bar graphs represent the mean±S.D. of 5-6 measurements. $^*p<0.05$, $^{**}p<0.01$, ns=not significant.
